# GLP-1 and Underlying Beneficial Actions in Alzheimer’s Disease, Hypertension, and NASH

**DOI:** 10.3389/fendo.2021.721198

**Published:** 2021-09-06

**Authors:** Qiu-Xuan Li, Han Gao, Yue-Xin Guo, Bo-Ya Wang, Rong-xuan Hua, Lei Gao, Hong-Wei Shang, Xin Lu, Jing-Dong Xu

**Affiliations:** ^1^Clinical Medicine of “5+3” Program, Xuanwu Hospital, Capital Medical University, Beijing, China; ^2^Department of Physiology and Pathophysiology, School of Basic Medical Sciences, Capital Medical University, Beijing, China; ^3^Department of Oral Medicine, Basic Medical College, Capital Medical University, Beijing, China; ^4^Eight Program of Clinical Medicine, Peking University Health Science Center, Beijing, China; ^5^Department of Biomedical Informatics, School of Biomedical Engineering. Capital Medical University, Beijing, China; ^6^Morphological Experiment Center, School of Basic Medical Sciences, Capital Medical University, Beijing, China

**Keywords:** GLP-1, Alzheimer’s disease, blood pressure, DPP-4, non-alcoholic steatohepatitis, signaling pathway

## Abstract

GLP-1 is derived from intestinal L cells, which takes effect through binding to GLP-1R and is inactivated by the enzyme dipeptidyl peptidase-4 (DPP-4). Since its discovery, GLP-1 has emerged as an incretin hormone for its facilitation in insulin release and reduction of insulin resistance (IR). However, GLP-1 possesses broader pharmacological effects including anti-inflammation, neuro-protection, regulating blood pressure (BP), and reducing lipotoxicity. These effects are interconnected to the physiological and pathological processes of Alzheimer’s disease (AD), hypertension, and non-alcoholic steatohepatitis (NASH). Currently, the underlying mechanism of these effects is still not fully illustrated and a better understanding of them may help identify promising therapeutic targets of AD, hypertension, and NASH. Therefore, we focus on the biological characteristics of GLP-1, render an overview of the mechanism of GLP-1 effects in diseases, and investigate the potential of GLP-1 analogues for the treatment of related diseases in this review.

## Highlights

- Apart from facilitating insulin secretion, additional effects of GLP-1 include anti-inflammation, neuro-protection, and reducing the accumulation of fat.
- There is a high consistency between AD and T2DM, both of which occur with insulin resistance (IR) in the brain.
- As the native GLP-1 has a short half-life, GLP-1 analogues have been produced to prolong effects and many of them could cross blood-brain barriers (BBB) such as Liraglutide (LG), lixisenatide, and exendin-4.
- GLP-1 analogues have proven utility in retarding diseases development in many animal and clinical trials, which are the potential and promising drugs to be utilized in AD, hypertension, and NASH.

## Introduction

With the assistance of prediction and decipherment of recombinant cDNA clones, it has been claimed that the anglerfish preproglucagon cDNA encodes a different glucagon-related peptide (1–3). Afterwards, two glucagon-related peptides, glucagon-like peptides 1 and 2 (GLP-1 and GLP-2), were recognized in the hamster (4), rat (5), and human (6) proglucagon sequence. In various experimental models, GLP-1, which is extracted from the intestines of humans and the porcine gut, can promote glucose-dependent insulin secretion (7–9). Based on this, GLP-1 is classified as an incretin hormone (10), promoting its use in the treatment of type 2 diabetes mellitus (T2DM). In the preceding 20 years, copious strategies for treating T2DM have been generated in various clinical and fundamental studies. However, apart from its role in improving glucose control, extensive effects of GLP-1 and its broad potential in pharmacology still remain to be explored. Other studies have shown that GLP-1 possesses neuroprotection functions along with enhancement of cognitive functions (11). Moreover, GLP-1 analogues, including Liraglutide (LG) and exendin-4, possess longer half-lives than native GLP-1 and provide prospects for broader clinical applications (12). Studies have also shown that LG possesses neuroprotection and anti-inflammatory effects, which could conceivably delay the progression of AD (13). As of right now, neuroprotective and anti-inflammatory effects of GLP-1 analogues have been investigated in AD, while more detailed actions of them still remain to be clarified. Additionally, numerous investigations found that blood pressure (BP) and heart rate (HR) increased transiently when normal rodents were given the acute infusion of GLP-1R agonists (14) while chronic injection of that reduces BP in mice studies (15). This decrease in BP was found in hypertensive patients with T2DM when they were given GLP-1R agonists over a long period of time (16). It is possible that the difference in injection speed and physiological status could account for these opposite BP alterations which were induced by GLP-1 and GLP-1R agonists. Along with that, GLP-1 has a close correlation with non-alcoholic steatohepatitis (NASH), which is characterized by hepatic inflammation and cell injury. NASH has currently been regarded as the main cause of the increased burden of hepatocellular carcinoma (17), but the molecular mechanism of it is still complex and multifactorial (18). Moreover, the use of GLP-1 analogues LG and Exendin-4 effectively reduced both their weight and liver fat in NASH patients (19). Based on the findings above, this review focuses on the biological characteristics and the underlying mechanistic effects of GLP-1 in AD, BP, and NASH.

## Biological Characteristics of GLP-1

**Figure 1:**
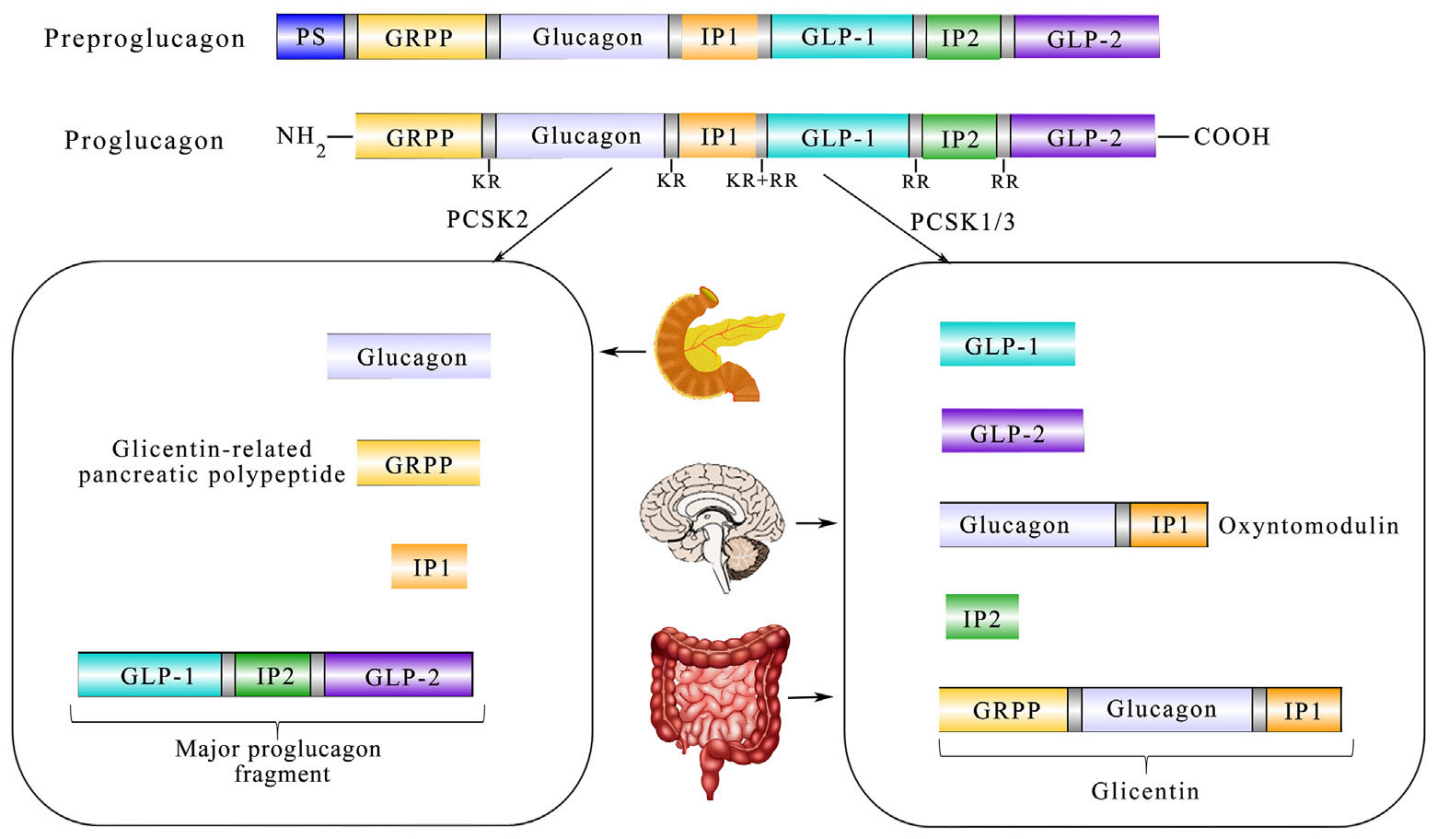
Diagram showing the relationship between translation and post-processing of glucagon precursors and its physiological processing pattern in the tissue. Coded by the preproglucagon gene, proglucagon is catalyzed by PCSK1/3 in the gut and brain and further processed to glicentin-related pancreatic polypeptide (GRPP) and oxyntomodulin (OXM), GLP-1, intervening peptide-2 (IP-2), and GLP-2. In the α-cells of the pancreatic islet, PCSK2 predominates and processes proglucagon to glucagon, GRPP, intervening peptide 1 (lP1), and a proglucagon fragment.

It is well-known that GLP-1 has manifold forms processed from proglucagon, such as GLP-1 (1–37), GLP-1 (7-36 amide), and GLP-1 (7–37) (20). Furthermore, proglucagon is cleaved from preproglucagon and is differentially processed *via* the prohormone convertase 2 (PCSK2) and prohormone convertase 1/3 (PCSK1/3) as shown in **Box 1**. In addition to intestinal L cells, pancreas α-cells and the nucleus tractus solitarii (NTS) are also sites of preproglucagon processing (24). The proglucagon gene (*Gcg*) encodes human’s preproglucagon and the constituent parts of the promoter of Gcg consist of four enhancers including G1, G2, G3, and G4 together with a cAMP response element (CRE_PG_). Although G2-G4 enhancers of rats could markedly activate the expression of more proximal human promoters, the comparable human sequences homologous to the G2–G4 enhancers are insufficient to activate reporter expression from the proximal rat promoter in islet and intestinal cells (25). In addition, CRE_PG_ could mediate transcriptional responses to physiological stimuli from the neuronal and dietary origin (26). As shown in **Figure 1**, the spliceosome of proglucagon includes glicentin, glicentin-related pancreatic polypeptide (GRPP), glucagon, oxyntomodulin (OXM), GLP-1, and GLP-2 (27).

Box 1 Proprotein convertases (PPCs) are a family of proteins responsible for other proteins activation and involved in many important biological processes, such as cholesterol synthesis.

PCSK1/3 and PCSK2, PPCs subtypes in humans, perform the proteolytic cleavage of prohormones to their intermediate forms (21).

Brain and intestine GCG + cells are discovered to express PCSK1/3 while PCSK2 is expressed in the pancreas (22, 23).

In 1966, Hopsu-Havu and Glenner discovered the existence of the enzyme dipeptidyl peptidase-4 (DPP-4) (28), encoded by the *DPP4* gene. A study has shown that high expression of DPP-4 is not only found in intestinal epithelial brush border, but also in endothelial cells (29). Owing to DPP-4 and renal elimination, native GLP-1 in human retain a short-lived half-life of around 1-2 minutes. For this reason, GLP-1 analogues were synthesized to prolong their short half-life. Meanwhile, DPP-4 cleaves the two active forms of GLP-1, GLP-1 (7-36 amide) and GLP-1 (7–37), into GLP-1 (9-36 amide) and GLP-1 (9-37), which have a relatively low affinity to GLP-1R and serve as the major circulating forms (30–32). These intact forms and inactive GLP-1 metabolites can be quickly cleared *via* renal elimination. By contrast, there is some laboratory evidence, indicating that kidneys are not the chief organs of the DPP-4 mediated metabolism of GLP-1 (33). Further studies in pigs revealed that a high degree of NH2-terminal degradation of GLP-1 occured in the hepatic portal system (34), concomitant with the evidence that DPP-4 is found in high concentrations in hepatocytes and endothelial cells (35, 36). There are two types of DPP-4, the membrane-spanning cell surface protein and the circulating protein (37). Pharmacological inhibition of DPP-4 using DPP-4 inhibitor sitagliptin could inhibit GLP-1 cleavage to maintain a higher concentration of GLP-1 in the blood circulation (29). This could induce insulin secretion from β cells of islet so as to improve glucose tolerance in normal and diabetic animals (38, 39).

## Tissue Distribution of GLP-1R

**Figure 2:**
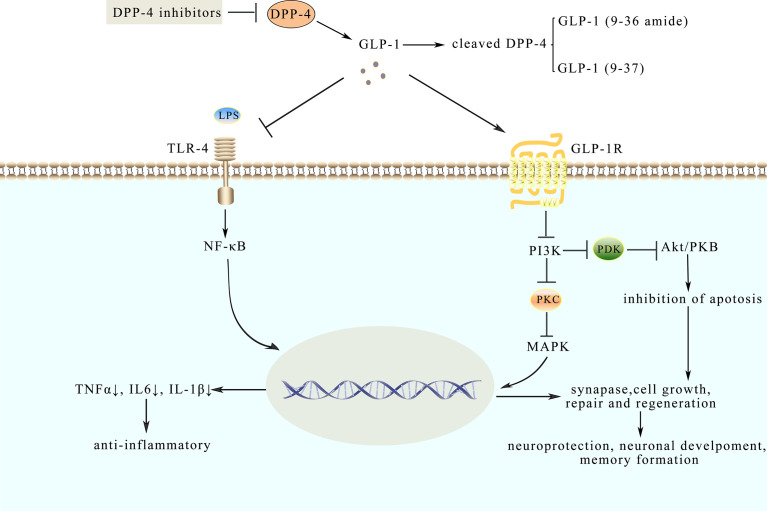
Model diagram of potential mechanisms of neuroprotective and anti-inflammatory effects of GLP-1. GLP-1 analogues like native GLP-1 have a longer half-life. Their effects are mediated through binding to GLP-1R, which could inhibit pathways such as the NF-κB pathway and MAPKs pathway. LPS, combined with TLR-4, activates the NF-κB pathway and triggers an inflammatory response while GLP-1 could inhibit the response to protect the synaptic plasticity. Besides, GLP-1 binds to GLP-1R to the active AMPK pathway involved in neuroprotection.

To gain further insights into the involvement of GLP-1 by binding to GLP-1R (40), GLP-1R’s presence was first confirmed and effects of the peptide on cAMP concentrations were investigated (9). However, the analysis of GLP-1R always confronted an obstacle due to the absence of antibodies that were sufficiently selective and available. Further, it is noteworthy that there was disputed information in the expression of GLP-1R in different cell types. Due to the lack of selective antibodies and application of specific anti-GPCR antibodies (41), the exact cellular localization of the GLP-1R remains equivocal (42). Notably, Novo Nordisk, a Danish pharmaceutical company, has recently developed the monoclonal antibody [MAb]3F52, which can specifically bind to the extracellular domain (ECD) Fab region of GLP-1R, and enabled the selective detection of GLP-1R in C57BL mice (43). Different from specific anti-GPCR antibodies, the monoclonal GLP-1R antibody could be used for specific and highly sensitive detection of GLP-1R in primate pancreas, kidney, lung, heart, gastrointestinal (GI) tract, liver, and thyroid. It is generally accepted that GLP-1R was detected in the β-cells, arterial walls of the kidney and lung, heart myocytes of the sinoatrial node, and the Brunner’s gland of the duodenum (43). Moreover, GLP-1 expressed in the liver has been reported, while no other publications showed its expression in hepatocytes (44). In terms of structure, GLP-1R is constituted by seven transmembrane helices (TMH) interconnected by intracellular loops, accompanied by a C-terminal intracellular domain and a large (w120 amino acid) N-terminal extracellular domain (ECD) (45). GLP-1 binds to GLP-1R in a complicated procedure, with GLP-1 peptide stably anchored in its position through an extensive network of interactions (40). In particular, recent studies identified the expression of GLP-1R in adipocytes (46, 47). The latest research proved that LG promotes pre-adipocytes differentiation and decreased fatty acid synthase (FASN) expression in differentiated adipocytes (47). In addition, LG could decrease lipogenesis in the liver in times of IR, driving the pathogenesis of non-alcoholic steatohepatitis (NASH) (48). Furthermore, some experiments demonstrated that LG could serve as an important intervention in NASH, which increased adipose insulin sensitivity (49). Moreover, PKA and ERK1/2 inhibitors can reverse the LG-induced FASN down-regulation. In GLP-1R^-/-^ mice, lack of GLP-1R expression in adipocytes caused a reduction in adipogenesis, through the induction of apoptosis in pre-adipocytes by inhibiting ERK, PKC, and AKT signaling pathways (50) (**Figure 2**). Among non-human primates (NHPs), the highest GLP-1 immunoreactivity is present in the hypothalamus, the area postrema, the NTS, and the dorsal vagal nucleus (51). In the human brain, GLP-1R mRNA is expressed in the cerebral mantle, the hypothalamus, the hippocampus, thalamus, putamen, and the dorsal pallidum (44). The high affinity and specificity could be showed in GLP-1 binding to GLP-1R, suggesting that GLP-1 may play a potential role in the central nervous system (CNS) through mediating some biological processes such as food intake and body weight (BM) (52). Actually, GLP-1 analogues have the ability to lower body weight through inhibition of food intake, which is related to peripheral GLP-1R signaling and central GLP-1R signaling. In 1996, it was reported that intracerebroventricular (ICV) of GLP-1 reduced food intake in fasted rats in a dose-dependent (53), suggesting that GLP-1 may act on central GLP-1R to produce satiation. Furthermore, exendin (9–39), a selective antagonist of GLP-1R, blocked the inhibitory effect of GLP-1 on food intake in fasted rats (53) with an increase in food intake and fat mass (54). Along with that, the anorectic effect vanished after administering LG and exendin-4 in GLP-1R KO mice (55). This suggested that central GLP-1 had a physiological role in regulating food intake in rats and GLP-1R may be necessary for the anorectic effect. Recent studies have also reported that the expression of hindbrain GLP-1R control food intake and BM in rats (56). Besides, infusion of exendin-4 into the 4th ventricle reduced food intake through reducing meal frequency in rats (57). Undoubtedly, central GLP-1R signaling is vital for the anorectic effect of GLP-1 analogues. In humans, satiety was increased and food intake was reduced after IV infusions of physiological doses of GLP-1 during meals (58, 59). Furthermore, the anorectic effects of IP injection of LG and exendin-4 were attenuated by subdiaphragmatic vagal deafferentation in rats, indicating that peripheral GLP-1 could inhibit food intake. In humans, the anorectic effect of GLP-1 was disappeared after truncal vagotomy (60), suggesting that vagal afferents are related to mediating the satiating effects of GLP-1. Despite the findings above, the mechanisms underlying GLP-1 inhibiting food intake and BM are still complex. A variety of evidence indicates that peripheral injection of GLP-1 analogues may act directly on the hypothalamus and hindbrain or the signal is transmitted to the hindbrain by vagal afferents (57). With that, central GLP-1R activation suppresses food intake and lower BM by enhancing phosphorylation of PKA and MAPK and decreasing the activity of AMPK in the NTS. Contrary to behavior therapy without them, multiple studies confirmed that LG and semaglutide could reduce BM and enhance weight loss maintenance (61). Furthermore, reducing weight effects of semaglutide is greater than LG (62). Therefore, it is worth anticipating that GLP-1 analogues are hopeful obesity drugs to control food intake, BM, and appetite.

## Dysfunction of GLP-1 and Diseases

### GLP-1 and AD

AD is the most common neurodegenerative disease with an incurable cognitive impairment, but recently hope is proposed due to the development of Biogen’s monoclonal antibody drug aducanumab (ADU) in the USA. ADU selectively binds to Aβ fibrils and soluble oligomers, which reduced amyloid plaques in a dose-dependent and time-dependent manner (63). However, it has not been proven that ADU has clinical benefits in treating cognitive dysfunction in AD (64). Despite the extensive studies in the past decades, the fundamental mechanism responsible for the development and progression of AD has not yet been fully elucidated. Recent experimental and clinical studies have shown that AD can be considered as a metabolic disorder corresponding with T2DM (65) and is referred to as Type 3 diabetes in some instances. Postmortem analysis has revealed that insulin resistance (IR) also occur in AD patients brain with significantly decreased expression of the insulin receptor, concomitant with the disease progression (66, 67) and indicated that the defects in insulin signaling are associated with AD pathogenesis (68). It is also worth noting that the correlations between T2DM and AD have been found in epidemiological studies (69). Along with that, the network meta-analysis demonstrated that the cognition of AD patients could be significantly improved after using anti-diabetic drugs (70). Convincing evidence has proved that AD is impacted by GLP-1 analogues through various mechanisms (71), such as IR, inflammatory cytokines, and oxidative stress, which supports and helps the proposed correlations (69). In addition, evidence from recent experimental studies suggests that most GLP-1 analogues can be injected peripherally and absorbed into the brain, indicating that they could cross the blood-brain barrier (BBB) and exert physiological influences directly on the human brain (72) (detail seen **Box 2**). These impacts include increasing neuron progenitor cells proliferation, prolonging potentiation in the hippocampus, improving learning, as well as reducing plaque formation and inflammation in the brain (73). Hence, from the experimental results mentioned above, it could be inferred that anti-diabetic incretin-related drugs may indirectly affect AD. It has also been confirmed that AD pathologic markers, oligomeric Aβ and Aβ plaque load, could be reduced by LG and microglial activation is decreased and memory behaviors are improved in APP_swe_/PS1_E9 (APP/PS1) mouse model after using LG (74). In addition, mice overexpressing GLP-1R in the hippocampus has shown an increase in neurite growth and improvement in spatial learning abilities (75). It has also been demonstrated in a randomized, placebo-controlled, double-blind study that LG improved glucose metabolism and cognition in AD patients (76). Furthermore, in streptozocin (STZ i.c.v) -induced AD rats (77) and APP/PS1 mice (78), DPP-4 inhibitors sitagliptin and saxagliptin were observed to have the ability to eliminate Aβ accumulation, clear abnormal phosphorylation of tau, and improve brain mitochondrial dysfunction probably through activation of AMPK in neuronal cells (66). Besides, a novel GLP-1/GIP receptor agonist DA5-CH could reverse STZ-induced working memory and spatial memory impairments in rats (79). The drug also decreased the expression of tauS596 protein and increased the expression of synapse-related proteins in the hippocampus (79). As mentioned above, insulin receptors and synapses which correlate to memory in the brain are reduced after amyloid-β oligomers (AβOs) are infused into the lateral cerebral ventricle of NHPs, while LG could provide partial protection and decrease levels of tau (80). The neuroprotective effects of LG may involve in activation of the PKA signaling pathway, indicating that activating GLP-1R is propitious to protect brain insulin receptors and synapses in patients with AD (80). As mentioned above, DPP-4 inhibitors can increase endogenous GLP-1 levels by suppressing GLP-1 degradation. With that, DPP-4 inhibitors have a lower risk than GLP-1R agonists in leading to hypoglycemia. Therefore, it is beneficial for their possible usage in treating AD (81). Currently, common DPP-4 inhibitors include gliptin, saxagliptin, linagliptin, vildagliptin, and sitagliptin. Studies showed that gliptin is helpful for improving cognition in AD. Vildagliptin and sitagliptin could prevent mitochondrial dysfunction in the brain and improve learning behavior in high-fat diet-induced IR rats (82). Besides, previous studies in mice models of AD showed that linagliptin could decrease Aβ accumulation, attenuate tau phosphorylation and inhibit neuroinflammation (83). However, clinical data are still not sufficient and available for the application of DPP-4 inhibitors in AD patients. Based on the analysis above, despite the requirement of more clinical evidence, existing data has indicated the potential prospect for the use of GLP-1 and its analogues in AD treatment in the future (81).

Box 2 Protein kinase C (PKC), as a family of protein kinase enzymes, plays a vital part in several signal transduction cascades and have the ability to regulate other proteins through the phosphorylation of hydroxyl groups of serine and threonine amino acid residues.

As we know, proteins involved in p-p38 and p-JNK are MAPKs pathways and p-AKT are all proteins belonging to PI3K/Akt pathway.

Various GLP-1 analogues include exendin-4, LG, lixisenatide, albiglutide, dulaglutide, semeglutide, taspoglutide and so on. And small peptide GLP-1 analogues have been demonstrated to cross BBB *via* peripheral administration, including LG, lixisenatide and exendin-4 (72).

Analyzing cerebrospinal fluid (CSF), ventricular fluid (VF), and postmortem brain tissue by means of multiplex bead-based ELISAs, the results of the experiment further suggested that there are fifteen cytokines whose levels are elevated in CSF and brain in the course of early-stage AD (84). However, pro-inflammatory mediators in VF and brain are suppressed during later stages of AD, indicating that neuro-inflammation-mediated neurodegeneration occurs mainly in the early or a specific stage of AD rather than the whole clinical course (68). It is widely known that inflammation is a common feature in metabolic diseases, spreading from peripheral tissue to the brain, thus resulting in cognitive dysfunctions (85). Consequently, scientists put forward the “gut-brain axis” hypothesis to explain the functional collaboration between gut homeostasis and cognitive dysfunctions (85). Moreover, previous studies (53), as well as recent researches approved that GLP-1 analogues influence CNS to promote satiation (86). In order to further confirm the role of the gut-brain axis in this regulation, MG1363-pMG36e-GLP-1 was constructed, which could directly express GLP-1. As discussed above, lipopolysaccharides (LPS) could prompt inflammation and amyloidogenesis in the brain, by inducing the disorder of TNF-a, IL-1β, and cyclooxygenase-2 (COX-2) (87). MG1363-pMG36e-GLP-1 decreased the escape latency in mice that are injected with LPS. It has also impaired the ability of spatial learning and memory compared with the control model mice. Consistently, supplementation of MG1363-pMG36e-GLP-1 could also offset the markedly enhanced level of TLR-4 expressing on glial cells induced by LPS (88). According to the previous reports, LPS can bind to the TLR-4 on the glial cells, activate the NF-κB pathway to trigger inflammatory responses, and subsequently facilitate the secretion of TNF-α and IL-6 (89). This can alter the normal balance in synaptic plasticity and lead to cognitive decline (90). Similarly, GLP-1 reduced the expression of p-p38, p-JNK, and p-AKT, as shown in **Box 2**, suggesting that GLP-1 suppresses inflammatory responses by inhibiting the MAPKs signaling pathway (88) as shown in **Figure 2**. Therefore, anti-inflammatory and neuroprotection effects might be exerted through inhibiting NF-κB and MAPKs signaling pathways (88). Based on the results mentioned above, GLP-1 related signaling pathways have been corroborated as promising strategies to prevent and treat AD.

### GLP-1 and Hypertension

GLP-1 analogues LG and exendin-4 have been investigated in multiple models of hypertension. Not only do the effects on BP differ between acute and chronic administration methods, it is also seemingly species-dependent. A short-term administration of LG results in an unchanged or slightly elevated BP while the long-term administration has the opposite effect. Regardless of distinctive administration methods, it was confirmed that the speedy decline in BP could be observed after LG or exendin-4 injection in response to angiotensin II (Ang II) infusion in the C57BL/6 mouse model while in *Glp1r* ^−/−^ mice BP was not affected (91). Studies have also found that *in vitro* application of LG in isolated blood vessel segments cannot lower blood pressure. In most of the acute infusion studies conducted in healthy adults, BP was unchanged. Current clinical data indicate that GLP-1R agonists reduce BP in diabetic patients (92) and the antihypertensive effect can also be observed in Dahl salt-sensitive rats (93), in Ang II -induced hypertension rats (15, 91) and SHRs rats (94). Collectively, these studies indicate that the antihypertensive effect of GLP-1 is mediated by its natriuretic effect on the kidneys, vasodilation on endothelial cells, and reduction of sympathetic activity on brain stem catecholamine neurons (95). Following healthy human subjects infusion with native GLP-1, diastolic blood pressure (DBP) showed a slight increase, concomitant with a related increase in muscle sympathetic nerve activities (96). This suggests that endogenous GLP-1 might have the ability to reduce BP. Notably, multiple clinical experiments show that systolic blood pressure (SBP) is decreased by GLP-1R agonists in T2DM hypertensive patients while DBP is less affected (92, 97, 98). Furthermore, numerous research studies have shown that long-term blockade of GLP-1R signaling by GLP-1R antagonist exenatide- (9–39) enhances SBP but doesn’t affect DBP in normotensive and hypertensive rats, suggesting the relatively weak correlation between GLP-1 and DBP (99). Additionally, further investigations proved that a blockade of GLP-1R could result in the GFR reduction and NHE3-mediated sodium reabsorption enhancement in the proximal tubule (100). Previous studies have also demonstrated that GLP-1R agonists exendin-4 (Ex4) induced diuresis and natriuresis by increasing GFR and inhibiting the main renal proximal tubule sodium reabsorption in normotensive rats (101, 102). As mentioned above, GLP-1R signaling plays a natriuretic role to modulate Na^+^ balance and prevents volume expansion (95). It is also worth noting that GLP-1’s cardioprotective effects act independently of weight loss with a higher expression level of cardio-protective genes, including *Akt, GSK3b, PPARD* and so on (103). After intravenous administration of native GLP-1 in healthy adults, the cardiac output increased (104). Furthermore, Ex4 treatment of isolated mouse cardiomyocytes increases the phosphorylation of AKT and ERK (105) and has positive effects on cardiomyocytes growth and survival (106). Opposite results were also found in isolated adult rat cardiomyocytes that contraction was not be accelerated after treated with GLP-1 (107), accompanied with a decreased left ventricular contractility (108). At the same time, GLP-1 enhanced the heart recovery after ischemia and improved left ventricular functions in the same study, suggesting that its effects on BP may depend on different physiological conditions. Moreover, decreased HR and cardiac contractility, increased thickness of the left ventricular wall and left ventricular developed pressure (LVDP) were also seen in GLP-1^-/-^ mice (109, 110). Given this, the performance and output of the heart were improved by GLP-1 in the post-ischemic heart while cardiac output was decreased by GLP-1 in non-pathological conditions (111). In this way, it can be seen that BP fluctuates due to different physiological states, which could exert an influence on the functions of GLP-1.

Furthermore, in most studies concerning the chronic effects of GLP-1 on BP, BP is not the primary or pre-established endpoint and most data comes from studies in T2DM. In several studies, consistent decrease of SBP and BP-lowering occurred in the early period independent of weight loss (112–114). At the same time, when GLP-1 is used in conjunction with other antihypertensive agents, its antihypertensive effect appears to be adjunctive and independent of the effects of other antihypertensive agents (115). Presently, a large number of experimental animals have been used to elucidate its mechanisms, but the pathways aren’t detailed enough to be clearly illuminated in humans (91). More specifically, it is ambiguous whether this same pathway works in humans. That is to say, it may be beneficial for better application of GLP-1 drugs with a comprehensive understanding of their capacity to selectively lower BP. GLP-1 drugs are promising treatment option in cardiovascular diseases which is often a co-morbidity in T2DM.

### Correlation Between GLP-1 and Non-Alcoholic Steatohepatitis

**Figure 3:**
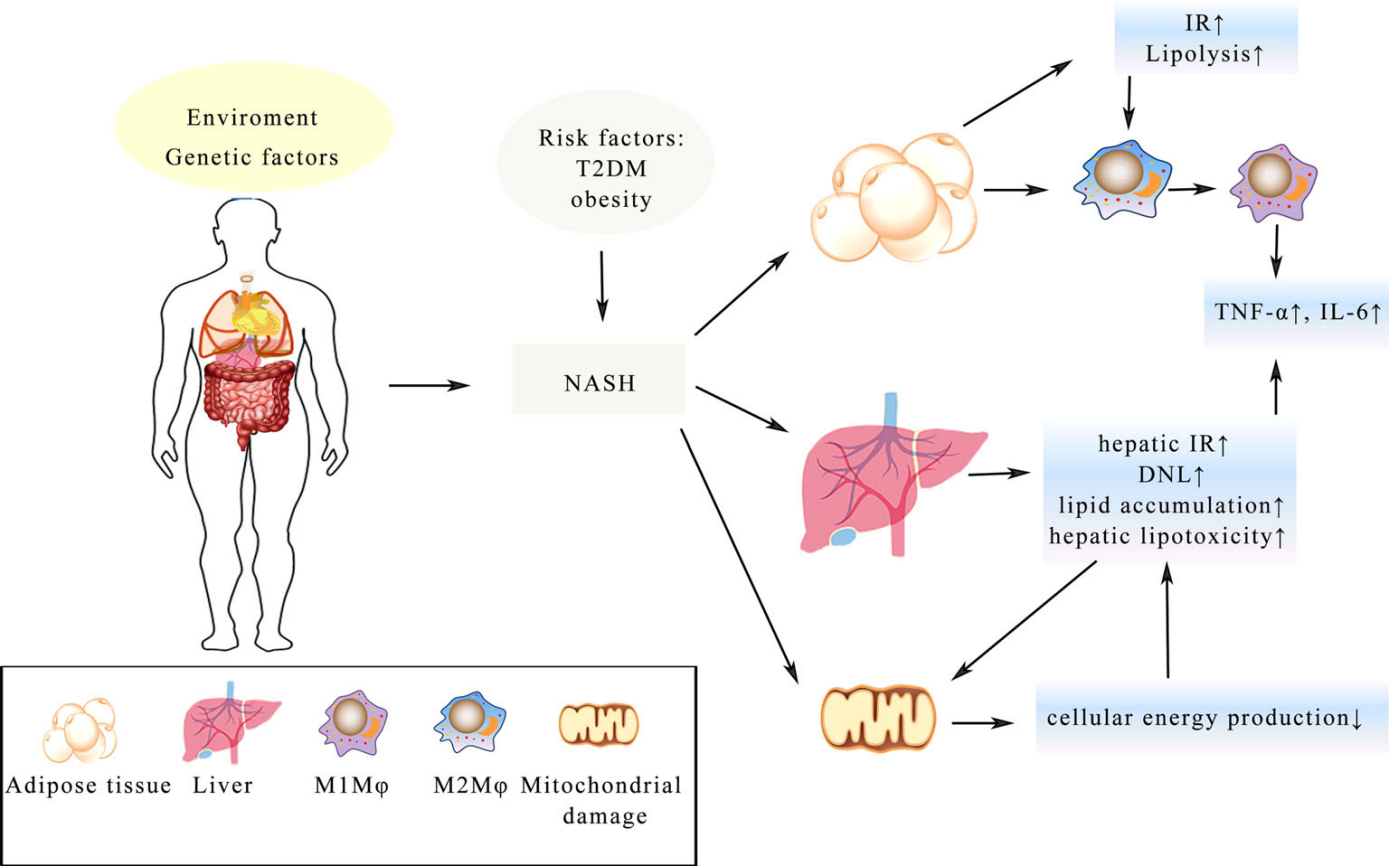
Illustration of the complex pathologic processes of NASH. T2DM and obesity increase the risk of NASH. IR in the adipose tissue and liver is a key driver, which leads to the imbalance of metabolism. Mitochondrial damage leads to less cellular energy production and adipose tissue hypertrophy, resulting in Mφ changing from the anti-inflammatory M2 Mφ state into the pro-inflammatory M1 Mφ. The process of all these changes will lead to enhance secretion of TNF-α, IL-6 and lipid accumulation in the hepatocyte, which eventually develop into fibrosis and cirrhosis.

There is currently a significant increase in the rate of patients with non-alcoholic fatty liver disease (NAFLD) with its prevalence rate reaching up to 40%. It has now surpassed viral liver disease to become the world’s most common liver disease, increasing the imminence of more basic and clinical research. A large number of studies have shown that the longer the disease lasts for, the more other serious diseases will develop such as liver cancer and liver failure. NAFLD, divided into two categories: non-alcoholic fatty liver (NAFL) and non-alcoholic steatohepatitis (NASH), is a group of diseases with different clinical manifestations and progression rates in individuals (116). With the overload supply of metabolic substrates, excessive fat accumulates in the hepatocytes, accompanied by gradual generation of potential toxic lipid species and the progressive increase in *de novo* lipogenesis (DNL) in NAFLD (117). Characterized by cell injury and inflammatory cell infiltration, NASH is regarded as a more aggressive form of NAFLD possibly progressing to cirrhosis and hepatocellular carcinoma with limited treatment options (18). Obesity and T2DM represent two of the major risk factors for NASH (118) and with a history of NASH, the possibility of suffering liver and cardiovascular diseases is greatly increased (119). Furthermore, IR in the liver and adipose tissue has been regarded as a crucial driver of NASH morbidity and mortality (48), while GLP-1 analogues have the ability to improve glycaemic control, lowering weight and activating liver enzymes in patients with T2DM. All these functions help promote GLP-1 an appealing therapy of NASH (120). Through promoting lipolysis in adipose tissue, IR is believed to exacerbate lipotoxicity, which is positive feedback further aggravating the imbalance of metabolism (121). Besides, macrophages (Mφ) in adipose tissue are also involved in IR and lead to the impairment of adipose tissue functions (122). Numerous studies have demonstrated that elevated fatty acid levels activate the TLR-4 signaling in adipose tissue, urging the polarization of Mφ to polarize from the anti-inflammatory M2 Mφ into the pro-inflammatory M1 Mφ (123). Furthermore, tumor necrosis factor-α (TNF-α) and IL-6 are produced by M1 Mφ, which are relevant components in the occurrence of IR (124). Besides, oxygen provision is deficient on account of hypertrophic adipose tissue and hypoxia is associated with fatty infiltration caused by M1 Mφ, which may be a potential mechanism of IR (122, 124). In the meantime, mitochondrial dysfunction is induced by hepatic IR and lipid accumulation and the lipid peroxidation mediated by cytochrome p450 2E1 boost the production of reactive oxygen and promote the degradation of mitochondria (125), which could further lead to the lack of cellular energy and metabolic intermediates accumulation, all these may lead to the secretion of pro-inflammatory cytokines, hepatocellular apoptosis and hepatic fibrogenesis, finally resulting in cirrhosis of the liver (125) (**Figure 3**).

To date, NASH is considered as a progressive form of NAFLD. Due to the complexity of NASH, no current treatment is approved for it (126) and most therapies remain to be traditional and focus on lifestyle intervention, which is difficult to maintain (127). Abundant evidence revealed that liver fibrosis is a key factor determining clinical outcomes in NASH patients (128). Pioglitazone and vitamin E are considered as possible treatment options while they have no effects on liver fibrosis (116). LG and semaglutide are proven to have beneficial effects on the histologic resolution of the disease (129, 130). Besides, the GLP-1R/GGCR dual agonist, Cotadutide, was shown to relieve steatosis, inflammation and fibrosis in both Ob/Ob NASH mice model and C57BI/6J NASH mice model (131). Several lines of evidence suggest that the potential roles of LG directly act on human liver cells *in vitro* to reduce steatosis by decreasing the level of DNL and increasing the fatty acid oxidation (132–134). Notably, after receiving LG, most patients showed improvements in steatosis and hepatocyte ballooning. Exciting experimental evidence available in patients with NASH suggests the potential of LG to reduce lipotoxicity by improving adipose tissue’s insulin sensitivities (49). After receiving LG, only a tiny portion of patients with NASH showed progression (129). Semaglutide, which is similar to LG, has been reported to reduce levels of alanine aminotransferase and markers of inflammation (135) with the levels of inflammation biomarkers being significantly lower after it treatment. Given the lack of liver GLP-1R expression (136), the potential mechanism of actions of GLP-1R agonists in NASH may be related to the indirect beneficial effects on body weight, IR and reduction in metabolic dysfunction, lipotoxic effects and inflammation (49, 137). LG and semaglutide treatment of NASH may be multifactorial, probably resulting from the accruing effects on losing weight and better control of glycaemic index (129). Moreover, Cotadutide was demonstrated to decrease fat accumulation in the liver and retard hepatic fibrogenesis developing in high-fat diet (HFD) and HFD/CCl4 mouse models (138, 139). Other studies show that Cotaduide could intervene and reverse NASH through improving metabolism in the liver including lipid and glucose metabolism *via* GCGR in mice, suggesting that Cotaduide may be a potential and viable therapeutic as a targeted drug intervention to reverse disease progression of NASH (131).

## Conclusions

To sum up, GLP-1 has extensive effects based on its biological characteristics through binding to GLP-1R. Apart from being used for treating T2DM, GLP-1 has a close relationship with AD and BP. Correspondingly, it is confirmed that GLP-1 analogues have neuroprotective and anti-inflammatory effects, which could alleviate learning and memory dysfunctions in the AD brain. GLP-1 is related to BP which depends on species, administration methods and physiological conditions. However, the principle of GLP-1 regulating BP is still not fully understood. Due to the ability to ameliorate IR and lipotoxicity in the pathogenesis of NASH, GLP-1R is a potential therapeutic target for NASH. In conclusion, GLP-1 provides a novel approach for treating AD, hypertension and NASH.

## Author Contributions

Q-XL and J-DX wrote the manuscript. HG, B-YW, and Y-XG designed the illustrations. H-WS, XL, and R-XH helped to analyze literature. J-DX and LG revised the manuscript. All authors contributed to the article and approved the submitted version.

## Funding

The research in the authors’ laboratory was supported by the National Natural Science Foundation of China Grant (No.8217140592, No.81673671) and Special National Key Research and Development Plan (No.2016YFC1306305 J-DX).

## Conflict of Interest

The authors declare that the research was conducted in the absence of any commercial or financial relationships that could be construed as a potential conflict of interest.

## Publisher’s Note

All claims expressed in this article are solely those of the authors and do not necessarily represent those of their affiliated organizations, or those of the publisher, the editors and the reviewers. Any product that may be evaluated in this article, or claim that may be made by its manufacturer, is not guaranteed or endorsed by the publisher.
